# Linking Targeted Pancreatic Cancer Genes With Metabolic Disorders: A Cross‐Species Translational Pathway

**DOI:** 10.1002/cam4.71775

**Published:** 2026-04-05

**Authors:** Dipanwita Nath, Caitlin Ditchfield, Joshua Price, Shivan Sivakumar, Simon W. Jones, Animesh Acharjee

**Affiliations:** ^1^ Department of Cancer and Genomic Sciences School of Medical Sciences, College of Medicine and Health, University of Birmingham Birmingham UK; ^2^ Department of Inflammation and Ageing MRC‐Versus Arthritis Centre for Musculoskeletal Ageing Research University of Birmingham Birmingham UK; ^3^ NIHR Birmingham Biomedical Research Centre University of Birmingham Birmingham UK; ^4^ Department of Immunology and Immunotherapy School of Infection, Inflammation and Immunology, College of Medicine and Health Birmingham UK; ^5^ MRC Health Data Research UK (HDR) Birmingham UK; ^6^ Centre for Health Data Research University of Birmingham Birmingham UK

**Keywords:** diabetes, metabolic inflammation, pancreatic ductal adenocarcinoma (PDAC), single‐cell RNA‐seq, translational oncology, unsupervised clustering

## Abstract

**Introduction:**

Pancreatic ductal adenocarcinoma (PDAC) remains one of the most lethal malignancies because of its typically late diagnosis and limited treatment options, with surgical resection being the primary intervention. Emerging studies have consistently reported associations between PDAC and metabolic dysfunctions, including obesity, chronic inflammation, and diabetes. In this study, we investigated the molecular interplay between PDAC‐associated genes and metabolic disorder pathways.

**Methods:**

We analysed publicly available bulk RNA‐Seq datasets from human and murine adipose tissues, complemented by single‐cell RNA‐Seq data from advanced‐stage PDAC. A set of key genes, ITGAM, PECAM1, CCL5, STAT1, STAT2, and CD44, was examined for expression patterns across datasets. Unsupervised clustering techniques were applied to single‐cell data to identify transcriptionally distinct populations. Functional analyses were conducted using KEGG pathway enrichment and STRING‐based protein–protein interaction networks. To experimentally validate transcriptomic findings, we performed ΔCT‐based quantitative PCR (qPCR) on human adipose tissue samples.

**Results:**

Gene expression analyses revealed significantly high expression of PDAC‐associated markers in both obese human and mouse models. Specific single‐cell clusters demonstrated transcriptional profiles linked to metabolic dysregulation in PDAC. Enrichment and network analyses implicated diabetic complication pathways and inflammatory signalling cascades. Experimental validation confirmed that genes such as ITGAM, CCL5, CXCL10, STAT1, and STAT2 were significantly upregulated in obese individuals compared to lean controls, underscoring a potential immunometabolic axis in PDAC pathophysiology.

**Conclusion:**

Our findings highlight a strong association between the upregulation of PDAC recurrence genes and the activation of metabolic pathways linked to obesity, diabetes, and inflammation. The consistent expression patterns across species suggest potential for developing targeted therapies to inhibit these metabolic pathways post‐pancreatic cancer resection, potentially reducing fatality.

**Abbreviations**
AGE: advanced glycation end‐products
AIF1: allograft inflammatory factor 1
AKT: AKT serine/threonine kinase (Protein Kinase B)
BMI: Body Mass Index
CCL3: C‐C motif chemokine ligand 3
CCL5: C‐C motif chemokine ligand 5
CD163: cluster of differentiation 163
CD44: cell surface adhesion receptor
CRP: C‐reactive protein
DEG: differentially expressed genes
DESeq2: differential gene expression analysis based onon the basis of the negative binomial distribution
DNA: deoxyribonucleic acid
ER: endoplasmic reticulum
EREG: epiregulin
FFA: free fatty acids
GEO: gene expression omnibus
GO: gene ontology
HCAR2: hydroxy carboxylic acid receptor 2
HLA‐DPA1: major histocompatibility complex, Class II, DP Alpha 1
HLA‐DPB1: major histocompatibility complex, Class II, DP Beta 1
HLA‐DQA1: major histocompatibility complex, Class II, DQ Alpha 1
HLA‐DQB1: major histocompatibility complex, Class II, DQ Beta 1
HLA‐DRA: major histocompatibility complex, Class II, DR Alpha
IAPP: islet amyloid polypeptide
IGF: insulin‐like growth factor
IGF‐1R: IGF‐1 receptor
IGFBP: IGF‐binding proteins
IL1A: interleukin 1 Alpha
IL1B: interleukin 1 Beta
IL‐6: interleukin‐6
IRS: insulin receptor substrate
ITGAM: integin subunit alpha M
KEGG: kyoto encyclopediaencyclopaedia of genes and genomes
KNN: K‐nearest neighbour
LST1: leukocyte specific transcript 1
MAPK: mitogen activated protein kinase
MIR3945HG: MIR3945 host gene
MNDA: myeloid cell nuclear differentiation antigen
mTOR: mechanistic target of rapamycin
NADPH: nicotinamide adenine dinucleotide phosphate hydrogen
NF‐κB: nuclear factor kappa‐light‐chain‐enhancer of activated B cells
OLR1: oxidised low‐density lipoprotein receptor 1
PCA: principal component analysis
PDAC: pancreatic ductal adenocarcinoma
PECAM1: platelet and endothelial cell adhesion molecule 1
PI3K: phosphoinositide 3‐kinase
PPAR: proliferator‐activated receptors
PPI: protein protein interaction
QC: quality control
qPCR: quantitative polymerase chain reaction
RAGE: receptors of advanced glycation end‐products
RNA‐Seq: RNA Sequencing
ROS: reactive oxygen species
S100B: S100 calcium binding protein B
SCFA: short chain fatty acids
SIGLEC1: sialic acid binding Ig like lectin 1
SNN: shared nearest neighbourneighbor
SPI1: Spi‐1 proto‐oncogene
SREBP: sterol regulatory element‐binding proteins
STAT1: signal transducer and activator of transcription 1
STAT2: signal transducer and activator of transcription 2
STRING: search tool for the retrieval of interacting genes/proteins
T1D: Type 1 diabetes mellitus
T2D: Type 2 diabetes mellitus
TAM: tumour‐associated macrophages
TME: tumour microenvironment
TNF‐α: tumour necrosis factor alpha
UMAP: uniform manifold approximation and projection

## 1 Introduction

Pancreatic ductal adenocarcinoma (PDAC) is characterised by a poor prognosis. The intricate relationship between PDAC and metabolic disorders, including obesity, chronic inflammation, and diabetes, suggests a shared pathophysiological pathway that may contribute to disease progression. Only 15% of PDAC patients are eligible for tumour‐removal surgery followed by adjuvant chemotherapy, which offers a 40% 5‐year survival rate. However, approximately 80% of these patients experience relapse [1]. Given the challenges of late diagnosis and limited therapeutic options, recurrent pancreatic cancer is typically fatal [1]. In our previous study [2], we conducted an integrative analysis of transcriptomic and proteomic networks associated with PDAC recurrence and identified a core set of genes—including ITGAM, PECAM1, CCL5, CXCL10, STAT1, STAT2 and CD44—that were consistently dysregulated across multiple metastatic sites. These genes were not only implicated in immune cell infiltration, inflammation, and vascular remodelling, but also showed centrality within the interaction networks, suggesting their potential role as key drivers of disease progression. Building upon those findings, the current study aims to explore whether these same markers are also relevant in the context of metabolic disorders such as obesity and diabetes, which are increasingly recognised as risk factors and modulators of PDAC pathophysiology. Through functional and expression profiling of these genes in metabolic contexts, we seek to delineate how systemic metabolic dysregulation drives pancreatic tumour recurrence, providing insights into potential molecular targets for therapeutic intervention. This mechanistic understanding underscores the need for translational studies to track metabolic parameters and improve recurrence prediction in pancreatic cancer patients [2]. Numerous epidemiological studies have established a positive correlation between obesity and the risk of pancreatic cancer. A meta‐analysis of cohort studies indicated that individuals with obesity (BMI ≥ 30 kg/m^2^) have a significantly increased risk of developing PDAC compared to those with a normal BMI (18.5–24.9 kg/m^2^) [3, 4]. The mechanisms through which obesity contributes to PDAC are multifaceted and are primarily linked to metabolic disruptions. A key factor is insulin resistance, which is commonly associated with obesity [5]. To counter insulin resistance, the body increases insulin production, leading to hyperinsulinemia [6]. Elevated insulin levels enhance the activity of the insulin‐like growth factor 1 (IGF‐1) signalling pathway, which plays a crucial role in cell growth and survival [7]. This pathway can stimulate the proliferation of pancreatic cancer cells, aiding tumour development and progression. Additionally, obesity often induces chronic inflammation and increased levels of adipokines (e.g., leptin and adiponectin), which can further increase cancer risk by promoting a pro‐tumourigenic microenvironment through elevated levels of pro‐inflammatory cytokines [8] such as TNF‐α, IL‐6, and CRP. NF‐κB activation by inflammatory cytokines [9] upregulates oncogenic transcriptional programs, including proliferative, anti‐apoptotic, and angiogenic pathways. Persistent NF‐κB signalling activation contributes to pancreatic ductal adenocarcinoma initiation and progression by sustaining tumour‐promoting pathways [10]. Thus, the combined metabolic dysregulation and chronic inflammation associated with obesity cooperatively enhance PDAC tumour growth and malignant progression [11]. Moreover, adipose tissue in obese individuals secretes high levels of Free Fatty Acids (FFA) [12, 13] which can induce oxidative stress and inflammation [5, 14], further promoting cancer cell proliferation, inhibiting apoptosis and metastasis.

Analysis of resectable PDAC cases [12] revealed that PDAC patients co‐morbid with diabetes presented with significantly larger tumours and experienced 50% higher postoperative mortality compared to non‐diabetic PDAC patients [14]. Diabetes mellitus, especially type 2 diabetes mellitus (T2DM), is intricately linked to pancreatic ductal adenocarcinoma (PDAC) [15], with epidemiological studies indicating a 1.5 to 2‐fold increase in PDAC risk among long‐standing diabetic patients [16]. New‐onset diabetes in older adults can sometimes precede the clinical diagnosis of PDAC, suggesting a bidirectional relationship where diabetes may both promote and be a consequence of PDAC [17]. Chronic hyperglycaemia in T2DM leads to increased glucose flux through glycolysis, causing metabolic stress and the production of advanced glycation end‐products (AGEs) [18, 19]. AGEs bind to their receptor (RAGE) on pancreatic cells [20], initiating signalling cascades that enhance proliferation and inhibit apoptosis, contributing to cancer cell survival and growth. Elevated glucose levels due to hyperglycaemia hence increase reactive oxygen species (ROS) production through mitochondrial dysfunction and enzymatic pathways like NADPH oxidase activation [20]. ROS induce DNA damage, genomic instability, and the activation of oncogenic pathways (e.g., MAPK and PI3K/AKT), fostering a carcinogenic environment in pancreatic tissues [21, 22]. In T2DM, insulin resistance in peripheral tissues results in compensatory hyperinsulinemia [7]. Elevated insulin levels bind to insulin receptors on pancreatic cells, promoting mitogenic signalling through the insulin receptor substrate (IRS) pathway [23]. Hyperinsulinemia increases the bioavailability of insulin‐like growth factor 1 (IGF‐1) by reducing IGF‐binding proteins (IGFBPs) [24]. IGF‐1 activates the IGF‐1 receptor (IGF‐1R), leading to the activation of downstream pathways such as PI3K/AKT and MAPK, which enhance cellular proliferation, inhibit apoptosis, and contribute to the oncogenic transformation of pancreatic cells [25]. T2DM often presents with dyslipidemia, characterised by elevated levels of circulating free fatty acids (FFAs) [13, 16] which contribute to lipid peroxidation and the generation of toxic metabolites, leading to endoplasmic reticulum (ER) stress and apoptosis resistance in pancreatic cells. FFAs can activate pathways such as peroxisome proliferator‐activated receptors (PPARs) and sterol regulatory element‐binding proteins (SREBPs), which modulate lipid synthesis and metabolism [26], potentially supporting the lipid demands of rapidly proliferating cancer cells. One study [27] revealed that PDAC impairs pancreatic β‐cell function and insulin secretion, likely through tumour‐derived factors including adrenomedullin and islet amyloid polypeptide (IAPP) [28]. These factors are proposed to induce systemic insulin resistance by disrupting peripheral insulin signalling, ultimately leading to hyperglycemia and diabetes onset [29]. This creates a vicious cycle: although metabolic disorders (particularly obesity and type 2 diabetes) elevate PDAC risk [3, 4], PDAC itself exacerbates glycemic control—an effect that persists even after tumour resection [12, 30]. Notably, this metabolic deterioration may worsen clinical outcomes by promoting PDAC recurrence and reducing overall survival. Hence, a cross‐species transcriptomic approach is essential to reveal the conserved immune—metabolic pathways that form the mechanistic link between systemic metabolic dysfunction and tumour recurrence biology in the pancreas.

## 2 Materials and Methods

### 2.1 Public Data Collection

For this study, a combination of publicly available Bulk RNA‐seq datasets, Single‐cell RNA‐seq datasets from NCBI‐GEO: Gene Expression Omnibus (https://www.ncbi.nlm.nih.gov/gds), and protein pathway datasets from KEGG: Kyoto Encyclopedia of Genes and Genomes (https://www.genome.jp/kegg/pathway.html) and Protein—Protein Interaction dataset from STRING Consortium (https://string‐db.org/cgi/about) were utilised. The objective was to investigate pancreatic ductal adenocarcinoma (PDAC) gene expression profiles and Protein Pathway analysis associated with metabolic disorders like obesity, diabetes, and inflammation. The datasets and relevant sample conditions are detailed in Table 1. Figure S1 highlights our systematic workflow throughout the study.

**TABLE 1:** List of datasets used in this study.

Dataset	Type of tissue	Organism
GSE246221	Liver adipose tissue	Human
GSE229053	Pancreatic Acinar cells	Mice
GSE263644	Cardiovascular System‐Associated Adipose Tissue	Human
GSE225224	Peripheral tissue	Mice
GSE212966	Pancreatic Cancer (Single cell RNA‐Seq dataset)	Human

### 2.2 Bulk RNA‐Seq Data Analysis

Publicly available datasets (GSE246221 [31], GSE229053 [12], GSE263644 [32], and GSE225224 [33]) were retrieved from the Gene Expression Omnibus (GEO) NCBI database, focusing on human and mouse tissue samples from healthy and obese individuals. These datasets were selected on the basis of predefined inclusion criteria requiring bulk RNA‐Seq tissue samples with clearly annotated healthy and obese groups, sufficient metadata quality, and representation across human and murine models to enable cross‐species translational comparison. More details of the PRISMA workflow of dataset selection are given in Figure S1. The raw gene expression counts were normalised using the DESeq2 package in R, which adjusts for differences in sequencing depth and other technical variables, ensuring that the observed differences in gene expression are biologically meaningful. The analysis centered on six target genes: ITGAM, PECAM1, CCL5, STAT1, STAT2, and CD44. Hence, this study was designed as a cross‐species translational analysis rather than an unbiased gene discovery investigation. In the present work, these genes were examined specifically to assess whether PDAC‐associated transcriptional programs are detectable within metabolically altered adipose tissues across species. These genes were selected on the basis of their relevance to pancreatic cancer recurrence sites, liver, and peritoneum [2]. To visualise the differential expression of these genes across healthy and obese conditions, box plots were generated using the ggplot2 package in R. These plots display the normalised expression levels of each gene across different groups: human healthy, human obese, mouse healthy, and mouse obese. Statistical significance was determined using the Wilcoxon rank sum test and interpreted in the context of cross‐dataset and cross‐species reproducibility rather than the magnitude of differential expression within any single dataset. Only comparisons with *p*‐values < 0.05 were considered significant and included in the plots.

### 2.3 Single‐Cell RNA‐Seq Data Analysis and Unsupervised Learning

#### 2.3.1 Data Acquisition and Preprocessing

The single‐cell RNA‐Seq dataset (GSE212966) [34, 35] of human pancreatic tumour tissue was retrieved from the GEO (Gene Expression Omnibus) repository. Initial quality control (QC) metrics were applied using the Seurat package in R to ensure data integrity. Cells were filtered on the basis of two main criteria: (1) Mitochondrial content: cells with a high percentage of mitochondrial gene expression were removed, as they may indicate cellular stress or damage, and (2) Gene counts: cells with either extremely low or high gene counts were excluded to avoid low‐quality cells or potential doublets.

#### 2.3.2 Normalisation and Scaling

Post‐QC, the data were normalised using the LogNormalize method in Seurat [36], which scales the gene expression values by the total expression, multiplies by a scale factor (typically 10,000), and logs the result. This method allows for comparison of gene expression levels across cells by adjusting for differences in sequencing depth.

#### 2.3.3 Dimensionality Reduction

To reduce the complexity of the high‐dimensional single‐cell RNA‐Seq data, Principal Component Analysis (PCA) was performed. PCA identifies the axes (principal components) that capture the maximum variance in the data [3], thus enabling the reduction of data to a lower‐dimensional space. The top principal components were selected on the basis of the Elbow Plot and used for downstream clustering analysis [37].

#### 2.3.4 Unsupervised Clustering With Seurat

Seurat's unsupervised clustering method was used to identify distinct cellular populations within the pancreatic tumour microenvironment. The clustering algorithm relies on the Shared Nearest Neighbour (SNN) modularity optimisation‐based clustering method [38]. A k‐nearest neighbour (KNN) graph was constructed on the basis of the Euclidean distance in the PCA space [39]. Each cell is connected to its nearest neighbours, forming a graph where nodes represent cells and edges represent the connections between them. The SNN graph is then divided into clusters by optimising a modularity function [38], which measures the density of connections within clusters compared to between clusters. The resolution parameter was adjusted to identify clusters at the appropriate granularity [39].

#### 2.3.5 Uniform Manifold Approximation and Projection (UMAP)

We used UMAP to visualise the high‐dimensional data in a two‐dimensional space. By using UMAP, we were able to preserve the local and global structure of the data, making it an effective tool for visualising the distinct clusters identified by Seurat [40]. The resulting UMAP plot displayed cells coloured by their assigned clusters, providing an intuitive visual representation of the cellular heterogeneity within the tumour microenvironment.

#### 2.3.6 Cluster Identification

To explore the expression patterns of marker genes across clusters, a heatmap was generated. Marker genes for each cluster were identified using Seurat's FindAllMarkers function, which performs a Wilcoxon rank‐sum test to identify genes significantly upregulated in each cluster compared to all other clusters [41]. The top marker genes determined by log fold change and *p*‐value were visualised in a heatmap, where rows represent genes, and columns represent clusters. The colour intensity corresponds to the normalised gene expression level, with higher expression depicted by brighter colours. Specific clusters of interest (e.g., Cluster 9) were highlighted within the heatmap to focus on their unique gene expression profiles using violin plots for our genes of interest across clusters, providing insights into the cellular characteristics and potential functional roles of these clusters within the tumour microenvironment.

### 2.4 Pathway Enrichment Analysis

To gain insights into the functional roles of the differentially expressed genes (DEGs) identified from the previous single‐cell Seurat analysis, we performed KEGG (Kyoto Encyclopedia of Genes and Genomes) pathway enrichment analysis. This analysis was specifically focused on the genes identified in Cluster 9, which exhibited a distinct expression profile and were hypothesised to play a crucial role in the inflammatory responses within the pancreatic tumour microenvironment. We extracted the genes from the specific cluster using Seurat's FindAllMarkers function. These DEGs were then categorised separately for human and mouse data. The list of DEGs was then subjected to pathway enrichment analysis using the clusterProfiler package in R. This package leverages the KEGG database to identify pathways significantly enriched by the input gene list. The enrichKEGG function was utilised for the analysis. Parameters were set to identify enriched pathways with an adjusted *p*‐value (p.adjust < 0.05), ensuring that only significantly enriched pathways were considered. The gene ontology (GO) terms were mapped to the KEGG pathways, and the analysis was conducted separately for both human and mouse datasets to identify species‐specific pathways. The enriched pathways were visualised using bar plots, where the x‐axis represents the number of genes involved in each pathway, and the y‐axis lists the pathways. The colour gradient of the bars represents the significance level (p.adjust) of each pathway, with more significant pathways shown in red. The pathways of interest, particularly those related to immune responses and inflammation, were highlighted for both human and mouse data.

### 2.5 Protein—Protein Interaction Network Analysis Using the STRING Database

The PPI network analysis was conducted to explore the functional interactions among proteins encoded by the genes identified as upregulated in Cluster 9 of the single‐cell RNA‐Seq analysis. This step was crucial for understanding how these proteins interact within the tumour microenvironment and how these interactions might contribute to disease mechanisms, particularly in the context of pancreatic ductal adenocarcinoma (PDAC) and metabolic disorders. Genes of interest for this analysis included those that were highly expressed in Cluster 9 on the basis of the Seurat differential expression analysis. These genes were: CD163, OLR1, IL1B, SIGLEC1, HLA‐DRA, HLA‐DPB1, HLA‐DQA1, HLA‐DQB1, HLA‐DPA1, SPI1, S100B, MNDA, EREG, LST1 and our targeted genes ITGAM, PECAM1, CCL5, STAT1, STAT2, CD44. The PPI network was constructed using the STRING database (version 11.5), a comprehensive resource that predicts protein–protein interactions on the basis of both experimental data and computational predictions. The list of selected genes was input into the STRING database for both human and mouse datasets separately. The interactions were set to a high confidence score (interaction score ≥ 0.7) to ensure the reliability of the network. STRING defines edges on the basis of multiple sources, including curated databases, experimental data, gene neighbourhood, gene fusions, co‐occurrence, and text mining. The analysis was performed to include only the interactions that were either experimentally determined or derived from curated databases, ensuring that the network reflects high‐confidence interactions. The network was clustered using the K‐means clustering algorithm to identify sub‐networks of proteins that might be co‐regulated or functionally related within Cluster 9. The clusters were annotated on the basis of known biological processes, pathways, and molecular functions. The network was visualised using Cytoscape software, where nodes represented proteins, and edges represented interactions. Nodes were colour‐coded on the basis of their presence in different signalling clusters, and the thickness of edges reflected the confidence level of each interaction. The network was displayed for both Mouse and Human datasets separately, showing species‐specific interactions and conserved network features across species. Hub proteins were identified by calculating the degree centrality of nodes within the network. Hub proteins are defined as those with the highest number of direct interactions (edges) with other proteins. These proteins play crucial roles in maintaining network stability and regulating key cellular processes [42]. Each signalling cluster within the PPI network was functionally annotated on the basis of the pathways and processes enriched within the cluster. The PPI network was interpreted in the context of PDAC, focusing on how perturbations in these protein interactions might contribute to disease mechanisms, particularly immune evasion, inflammation, and tumour progression. The identification of hub proteins provided insights into potential therapeutic targets that could disrupt disease‐associated networks.

### 2.6 Patient Recruitment and Sample Collection

To validate inflammatory gene signatures identified in PDAC‐metabolic networks, we analysed synovium and adipose tissue from osteoarthritis (OA) and healthy control patients, with either obese or with normal weight adiposity. OA tissues were selected as a clinically accessible model of chronic low‐grade inflammation and metabolic dysregulation, mirroring key features of the PDAC tumour microenvironment.

Synovium and subcutaneous adipose tissues were collected from OA patients or from neck‐of‐femur fracture patients, without OA, who were also undergoing total joint replacement surgery (NRES 17/SS/0456, 16/SS/0172 and 14/ES/1044) at the Royal Orthopaedic Hospital, Birmingham (United Kingdom) and Russell's Hall Hospital, Dudley (United Kingdom). Patients were considered normal‐weight if their BMI was 18.5–24.9 kg/m^2^ and obese if their BMI was 25.0–29.9 kg/m^2^ at the time of surgery.

### 2.7 RT‐qPCR and Statistical Tests

Total RNA was extracted from human OA synovium (*n* = 15 normal‐weight, *n* = 18 obese), human healthy control synovium (*n* = 10 normal‐weight neck‐of‐femur fracture), and human OA adipose tissue (*n* = 7 normal‐weight, *n* = 7 obese). Total RNA was extracted using a RNeasy Mini kit according to manufacturer's instructions (Qiagen). The quality and quantity of RNA were determined by Nanodrop One (Thermofisher); all isolated RNA samples had A260/A280 ratios of 1.8–2.0. mRNA levels of ITGAM, PECAM, CCL5, CXCL10, STAT1, STAT2, and GZMB were determined by RT‐qPCR, relative to the housekeeping gene GAPDH, using the iTaq Universal One‐Step RT‐qPCR Kit (Bio‐Rad) and gene‐specific primers (Thermofisher; assay IDs: ITGAM Hs00167304_m1, PECAM1 Hs01065279_m1, CCL5 Hs00982282_m1, CXCL10 Hs00171042_m1, STAT1 Hs01013996_m1, STAT2 Hs01013115_g1, GZMB Hs00188051_m1). Data were acquired using a Bio‐Rad CFX Opus, and ΔCT was calculated (housekeeping CT—gene of interest CT).

Data normality was assessed using Kolmogorov–Smirnov tests. Synovium data were analysed with one‐way ANOVA (Tukey's post hoc) for normally distributed data or Kruskal–Wallis (Dunn's post hoc) for non‐normal distributions, whereas adipose tissue data were evaluated using independent samples *t*‐tests (parametric) or Mann–Whitney U tests (non‐parametric).

**FIGURE 1:**
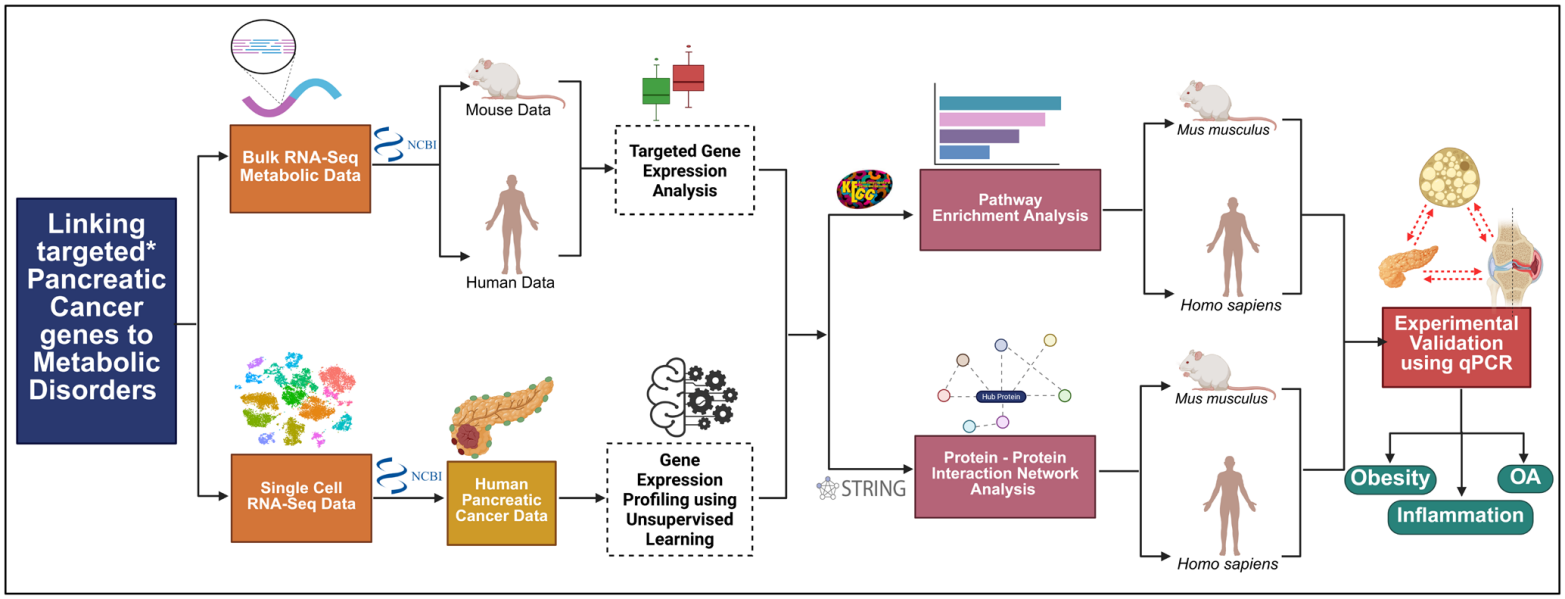
Overall workflow of the study. Targeted* genes are ITGAM, PECAM1, CCL5, STAT1, STAT2, and CD44.

The overall workflow is shown in Figure 1.

## 3 Results

### 3.1 Bulk RNA‐Seq Data Analysis

**FIGURE 2:**
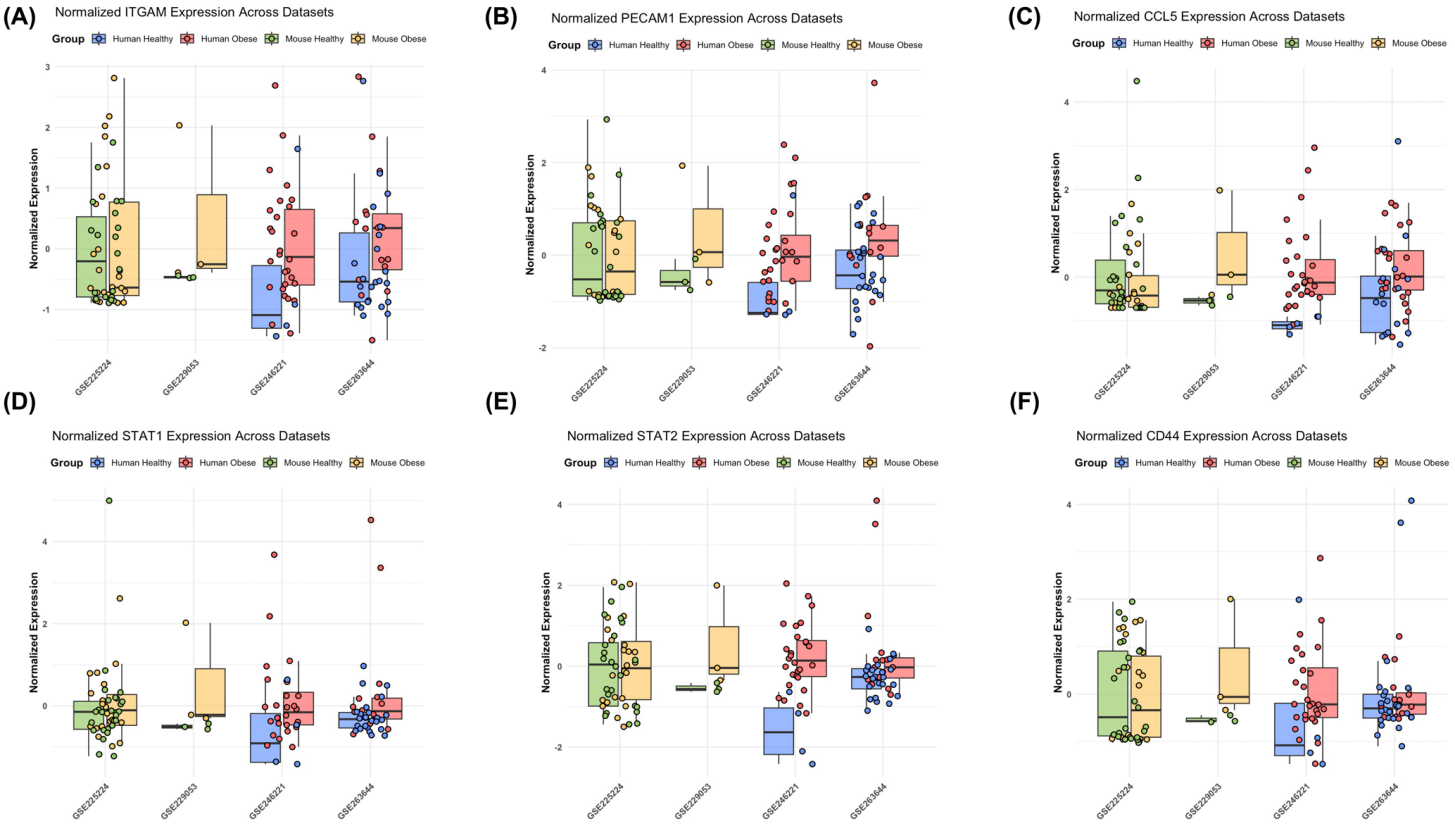
(A–F): Targeted gene (ITGAM, PECAM1, CCL5, STAT1, STAT2, and CD44) expression plots across different samples showing comparatively higher expression levels in metabolically disordered (obese) mice and humans compared to healthy individuals. Plots with *p* < 0.05 are only considered. Overlapping jittered points reflect individual sample variability and were retained to preserve consistent visualisation across datasets.

Figure 2 showcases the normalised expression levels of ITGAM, PECAM1, CCL5, STAT1, STAT2, and CD44 across the healthy and obese conditions in both human and mouse bulk RNA‐seq datasets. ITGAM expression (Figure 2A) is notably higher in obese mice and humans compared to their healthy counterparts, particularly in the GSE263644 human dataset and GSE225224 mouse dataset. This suggests a consistent upregulation of ITGAM in obesity, implicating its role in inflammation and immune cell recruitment in adipose tissue [43, 44]. PECAM1 (Figure 2B) also shows elevated expression in obese individuals in both species, with the GSE229053 mouse dataset showing a significant increase compared to healthy controls. This highlights PECAM1's potential involvement in endothelial cell function and inflammation in obesity [45]. CCL5 expression (Figure 2C) is significantly higher in obese conditions, particularly in the GSE225224 and GSE263644 datasets. The elevated levels of CCL5 in obese individuals suggest its role in recruiting immune cells to adipose tissue, promoting inflammation [46]. STAT1 expression (Figure 2D) is markedly higher in obese individuals, especially in the GSE229053 dataset for mice and the GSE263644 dataset for humans. This increase indicates a possible involvement of STAT1 in the inflammatory response associated with obesity [47]. Similar to STAT1, STAT2 expression (Figure 2E) is elevated in obese conditions, with significant differences observed in the GSE229053 mouse dataset and GSE263644 human dataset. STAT2 may contribute to the upregulation of immune responses in obesity [48]. CD44 (Figure 2F) shows a consistent increase in expression in obese individuals across the datasets, particularly in the GSE263644 human dataset. This finding suggests that CD44 may be involved in the inflammatory and immune responses seen in obesity, potentially through its role in cell adhesion and migration [49]. Overall, the results demonstrate that the selected target genes (ITGAM, PECAM1, CCL5, STAT1, STAT2, and CD44) are upregulated in both human and mouse models of obesity, indicating their significant roles in the inflammatory and immune processes associated with obesity‐related metabolic disorders.

### 3.2 Single‐Cell RNA‐Seq Data Analysis and Unsupervised Learning

**FIGURE 3:**
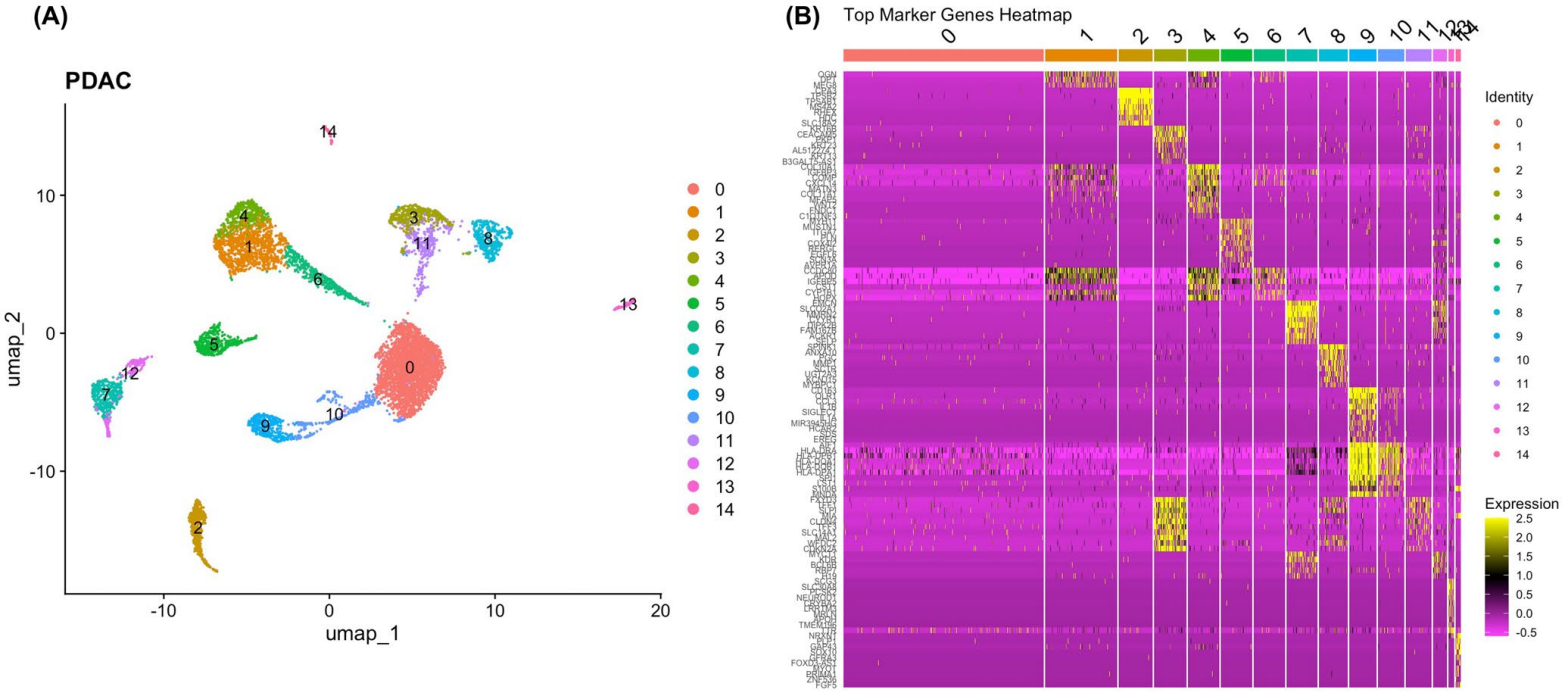
(A) Seurat Clusters of single cell analysis of the human pancreatic tumour microenvironment showing distinct clusters numbered from 0 to 14. (B) Heatmap showing top marker genes across all clusters. Cluster 9 is specifically highlighted in the heatmap plot. Dataset used: GSE212966.

The UMAP plot (Figure 3A) reveals cellular heterogeneity in the Pancreatic Tumour Microenvironment (TME). It demonstrates the identification of 15 distinct clusters within the human pancreatic tumour microenvironment, each represented by a different colour. These clusters were identified using unsupervised clustering methods in Seurat, revealing the diverse cellular populations present in the tumour tissue. Cluster numbering ranges from 0 to 14, with each cluster potentially representing different cell types or states within the tumour microenvironment. The heatmap (Figure 3B) highlights differential gene expression patterns of the top marker genes across all clusters. Cluster 9, specifically highlighted in the heatmap, shows a unique expression profile with upregulation of several marker genes, including CD163, OLR1, CCL3, IL1B, SIGLEC1, IL1A, MIR3945HG, HCAR2, EREG, AIF1, HLA‐DRA, HLA‐DPB1, HLA‐DQA1, HLA‐DQB1, HLA‐DPA1, SPI1, LST1, S100B, and MNDA. On the basis of established canonical markers, including CD163, LST1, AIF1, SPI1, and HLA class II genes, Cluster 9 was inferred to be enriched for myeloid‐lineage immune cells, consistent with macrophage/monocyte‐like populations within the tumour microenvironment. The differential expression analysis reveals that these marker genes are not uniformly expressed across all clusters, underscoring the cellular heterogeneity within the tumour. This heterogeneity is crucial for understanding the complex interactions and functional states of cells within the pancreatic tumour microenvironment, which may have implications for disease progression and therapeutic response.

**FIGURE 4:**
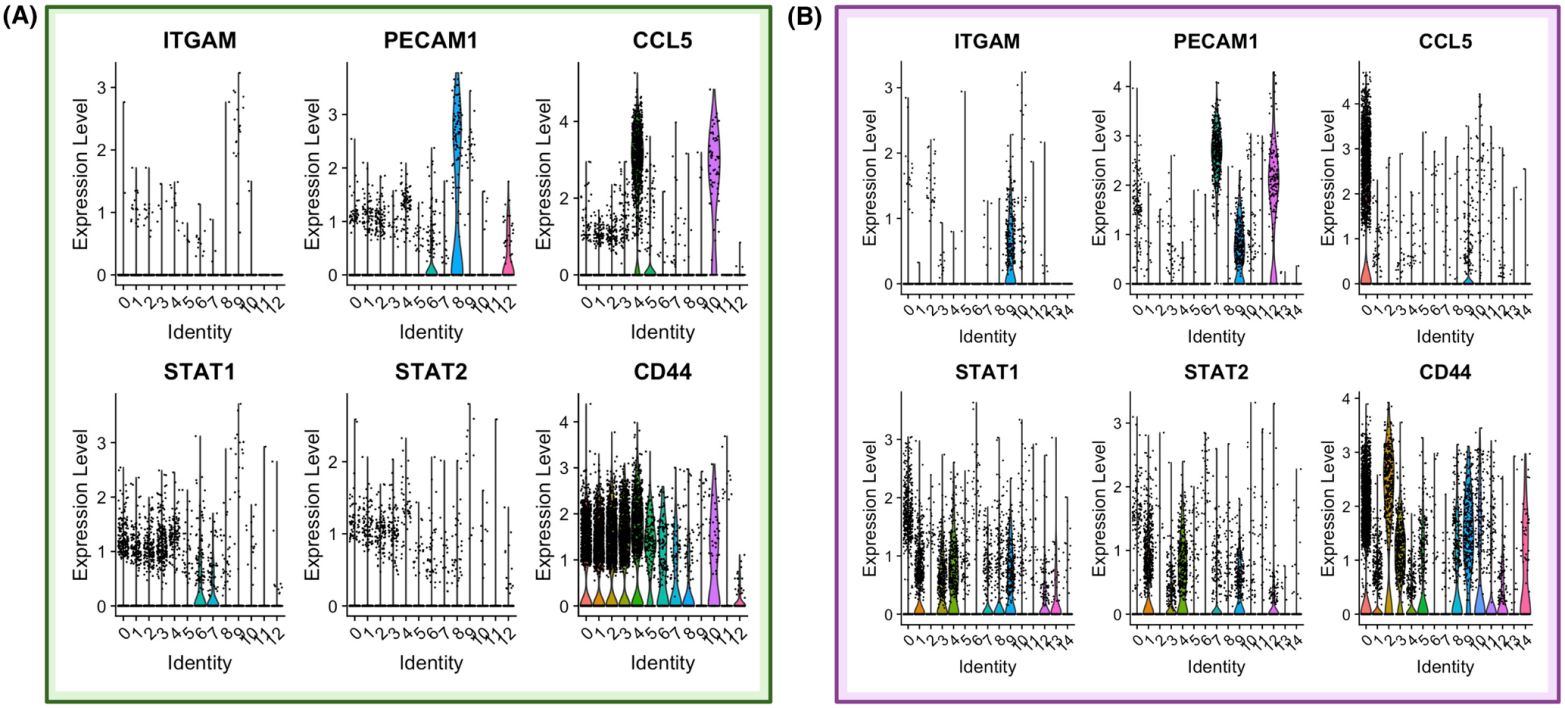
Violin plots showing the expression levels of genes of interest (ITGAM, PECAM1, CCL5, STAT1, STAT2, and CD44) across different clusters in the human pancreatic tissue, (A) non‐cancerous (control) and (B) cancerous. STAT1, STAT2, and CD44 have shown quite consistent expressions across all clusters. However, genes ITGAM, PECAM1, and CCL5 are highly expressed in clusters 8, 9, and 0 in cancerous compared to noncancerous. Identity signifies Cluster identities on the horizontal axis. Dataset used: GSE212966.

The violin plots in Figure 4 represent log‐normalised per‐cell expression values, where distribution shape reflects both expression magnitude and the proportion of expressing cells within each cluster. These results suggest that these clusters, particularly Cluster 9, are enriched in immune cell populations of myeloid origin that are transcriptionally active within the tumour STAT1, STAT2, and CD44 showed relatively consistent expression across all clusters, indicating their potential roles in maintaining baseline immune functions. In contrast, cancerous tissue (Figure 4B) exhibited higher expression of ITGAM, PECAM1, and CCL5 in clusters 8, 9, and 0, suggesting their involvement in the inflammatory and immune responses associated with pancreatic cancer progression. Especially cluster 9 in the cancerous tissue showed significant upregulation of genes involved in immune signalling, such as ITGAM (associated with macrophage activation) [44, 50], PECAM1 (involved in leukocyte migration) [45, 51, 52], and CCL5 (a chemokine linked to inflammatory responses) [46]. These results suggest that these clusters, particularly Cluster 9, are enriched in immune cell populations of myeloid origin that are transcriptionally active within the tumour immune microenvironment. The upregulation of ITGAM and CCL5, in particular, points to their roles in metabolic dysregulation within the tumour microenvironment. ITGAM is implicated in lipid metabolism and obesity‐related inflammation [44], whereas CCL5 is known to modulate glucose homeostasis and insulin sensitivity [46], linking these genes to diabetes and metabolic syndrome. The differential expression of these genes in cancerous versus non‐cancerous pancreatic tissue confirms the impact of metabolic alterations in pancreatic cancer, potentially contributing to disease progression and low survival.

### 3.3 Pathway Enrichment Analysis

**FIGURE 5:**
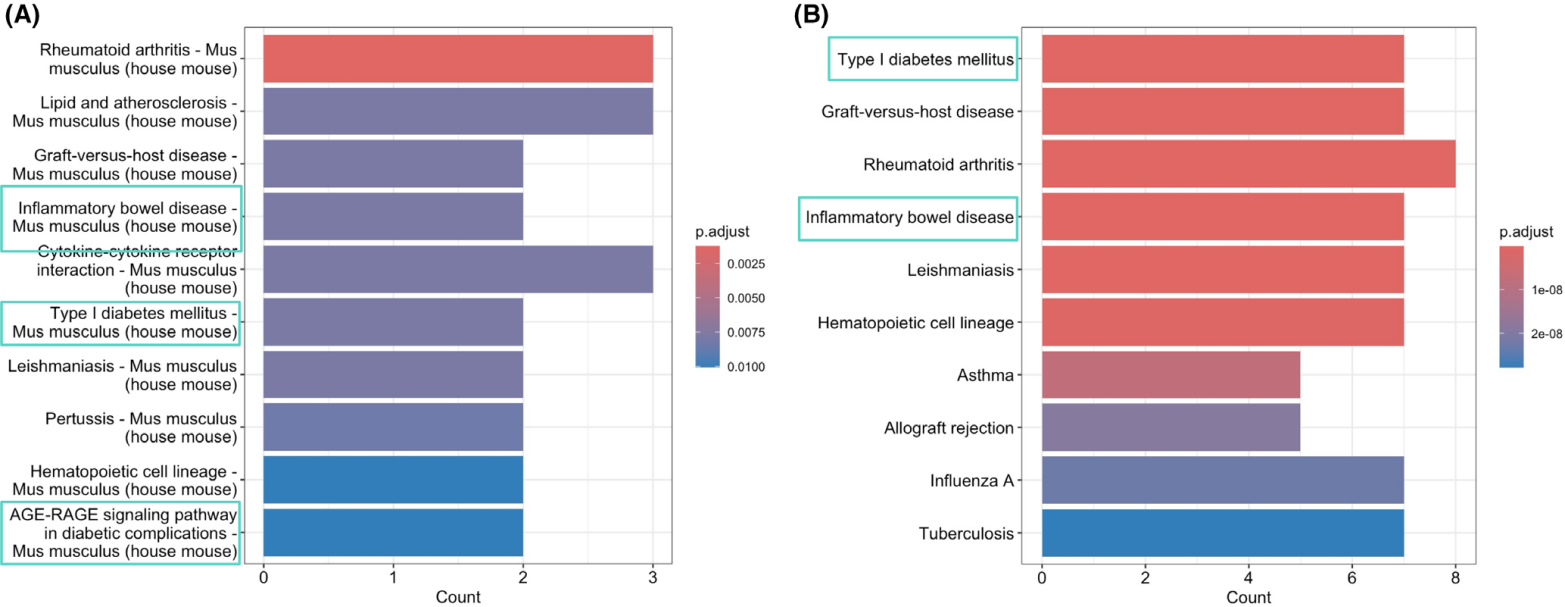
KEGG pathway enrichment analysis of genes of highlighted cluster 9 and predefined PDAC‐associated genes in (A) Mouse (B) Human showing Type‐I Diabetes, Inflammatory Bowel Disease, and AGE‐RAGE signalling pathway in diabetic complications to be enriched among others in both organisms.

Pathway enrichment analysis using the KEGG database identified several critical signalling pathways associated with the genes ITGAM, PECAM1, CCL5, STAT1, STAT2, and CD44 in both mouse and human datasets, as described in Figure 5. A total of 11 pathways were significantly enriched, with immune‐ and inflammation‐related pathways being predominant (Figure 9). Figure 5A shows the KEGG pathway enrichment results for the mouse dataset. Notably, the Rheumatoid arthritis pathway was the most enriched, indicating its role in autoimmune responses within the tumour microenvironment. Other significantly enriched pathways included Lipid and atherosclerosis, Graft‐versus‐host disease, Inflammatory bowel disease, Cytokine‐cytokine receptor interaction, and Type I diabetes mellitus. The presence of pathways such as AGE‐RAGE signalling in diabetic complications further suggests that the targeted genes may be involved in metabolic disorders alongside immune dysfunction in mice. Figure 5B displays the results of the KEGG pathway enrichment for the human dataset. Similar to the mouse data, the human genes were also significantly enriched in immune and inflammatory pathways. The most enriched pathways were Type I diabetes mellitus, Graft‐versus‐host disease, and Rheumatoid arthritis, highlighting the potential involvement of Cluster 9 in autoimmune conditions within the pancreatic tumour microenvironment. Pathways like Inflammatory bowel disease, Leishmaniasis, Haematopoietic cell lineage, and Asthma were also enriched, suggesting a diverse range of immune and inflammatory activities driven by Cluster 9 in human pancreatic cancer. The analysis also revealed significant enrichment in pathways such as Allograft rejection and Influenza A. The KEGG pathway enrichment analysis [53] for both mouse and human data consistently highlighted immune and inflammatory pathways, suggesting a conserved functional role for our targeted genes across species. The identification of similar pathways in both species strengthens the evidence that Cluster 9 proteins contribute to immune‐related activities within the pancreatic tumour microenvironment. The KEGG pathway enrichment analysis provided strong evidence that the targeted genes are significantly involved in pathways related to metabolic disorders: obesity, diabetes, and inflammation in both human and mouse. Detailed information about the pathway enrichment is provided in Figure S2, highlighting proteins in respective pathways, which were prioritised for display on the basis of their mechanistic relevance to immune‐metabolic and PDAC‐associated signalling axes.

### 3.4 Protein—Protein Interaction Network Analysis

#### 3.4.1 Mouse Network Overview

**FIGURE 6:**
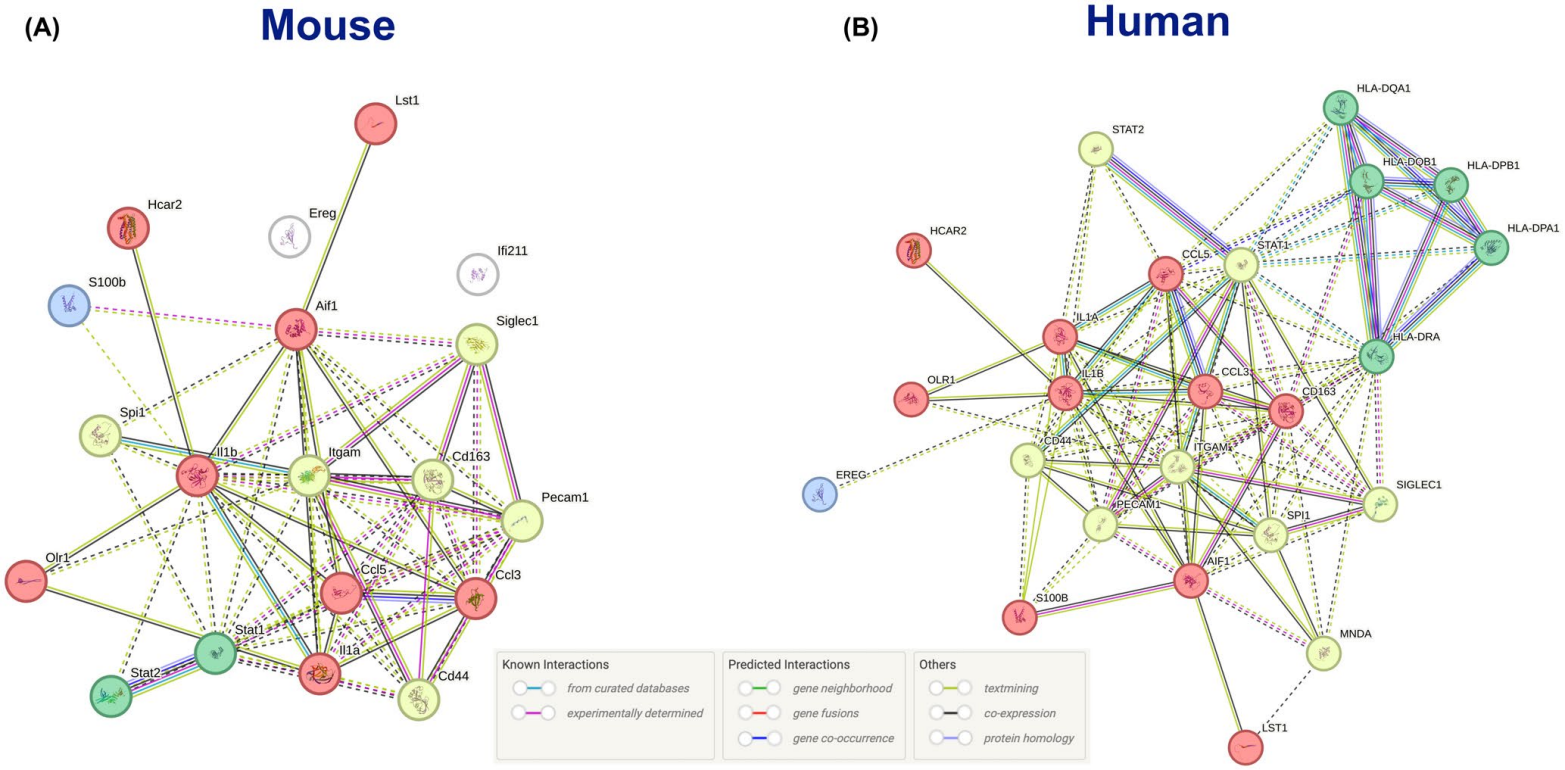
Protein ‐ Protein Interaction Network Analysis from the STRING database in (A) Mouse (B) Human highlighting genes from Seurat cluster 9 of the single cell analysis. Dotted lines suggest edges between clusters by the K‐means of their centroids. Cluster red suggests interleukin signalling in humans and lipopolysaccharide‐mediated signalling in Mouse. Cluster yellow suggests microglial cell‐mediated cytotoxicity in both organisms. Cluster green suggests Peptide antigen assembly with MHC class II protein complex in humans and interferon signalling in mice, and cluster blue suggests epiregulin (EREG) signalling pathway in humans and beta chain subunit in mice.

Figure 6A shows the PPI network for the mouse dataset, focusing on metabolic pathways. Key clusters include the Obesity Cluster involving ITGAM, CD163, and OLR1, which is enriched in pathways related to lipid metabolism and inflammation. These proteins are central to the regulation of obesity‐related metabolic disorders, particularly in the context of adipose tissue inflammation and macrophage activation. Next is the Diabetes Cluster featuring STAT1 and HLA‐DRA. This cluster is linked to insulin resistance and glucose metabolism. The proteins in this cluster are involved in the immune‐mediated dysregulation of metabolic processes that are characteristic of diabetes. Finally, the IBD Cluster encompasses IL1b and CCL5, which are involved in chronic inflammation and immune responses, key factors in the pathophysiology of IBD. The hub proteins in the mouse network include ITGAM, STAT1, and IL1b. These proteins are critical regulators of metabolic processes and are potential targets for modulating disease pathways in obesity, diabetes, and IBD.

#### 3.4.2 Human Network Overview

Figure 6B presents the human dataset's PPI network with important clusters, such as the Obesity Cluster that involves ITGAM, CD163, and OLR1, mirroring the mouse data and highlighting the conserved role of these proteins in lipid metabolism and inflammation. The human network emphasises the role of these proteins in obesity‐related metabolic diseases. The Diabetes Cluster with STAT1 and HLA‐DRA proteins that are key in immune responses that affect glucose metabolism and insulin sensitivity. The IBD Cluster involves IL1B and CCL5, proteins that are central to chronic inflammation and immune dysregulation as seen in IBD. The Human Hub proteins in the network include ITGAM, STAT1, and IL1B. These proteins represent critical nodes in the network that influence metabolic pathways related to obesity, diabetes, and IBD. We can also see the involvement of epiregulin (EREG) signalling, a growth factor involved in cancer progression, with S100B and EREG as central nodes.

#### 3.4.3 Cross‐Species Translational Overview

The PPI networks for both species show a strong conservation in the role of key metabolic pathways, particularly those involving lipid metabolism, insulin signalling, and chronic inflammation. The overlap in metabolic pathways between mouse and human datasets supports the relevance of these findings for understanding how metabolic conditions influence PDAC progression. The presence of hub proteins such as ITGAM and STAT1 across both species highlights their potential as universal therapeutic targets in modulating immune and inflammatory responses within the tumour microenvironment. The differences in network topology between mouse and human datasets provide insights into species‐specific mechanisms of immune regulation in PDAC, which may have implications for the translation of therapeutic strategies from preclinical models to human patients [3, 54, 55]. Figures S3 and S4 provides further information highlighting individual networks for each gene of interest according to the Human Protein Atlas.

### 3.5 Results of RT‐PCR

**FIGURE 7:**
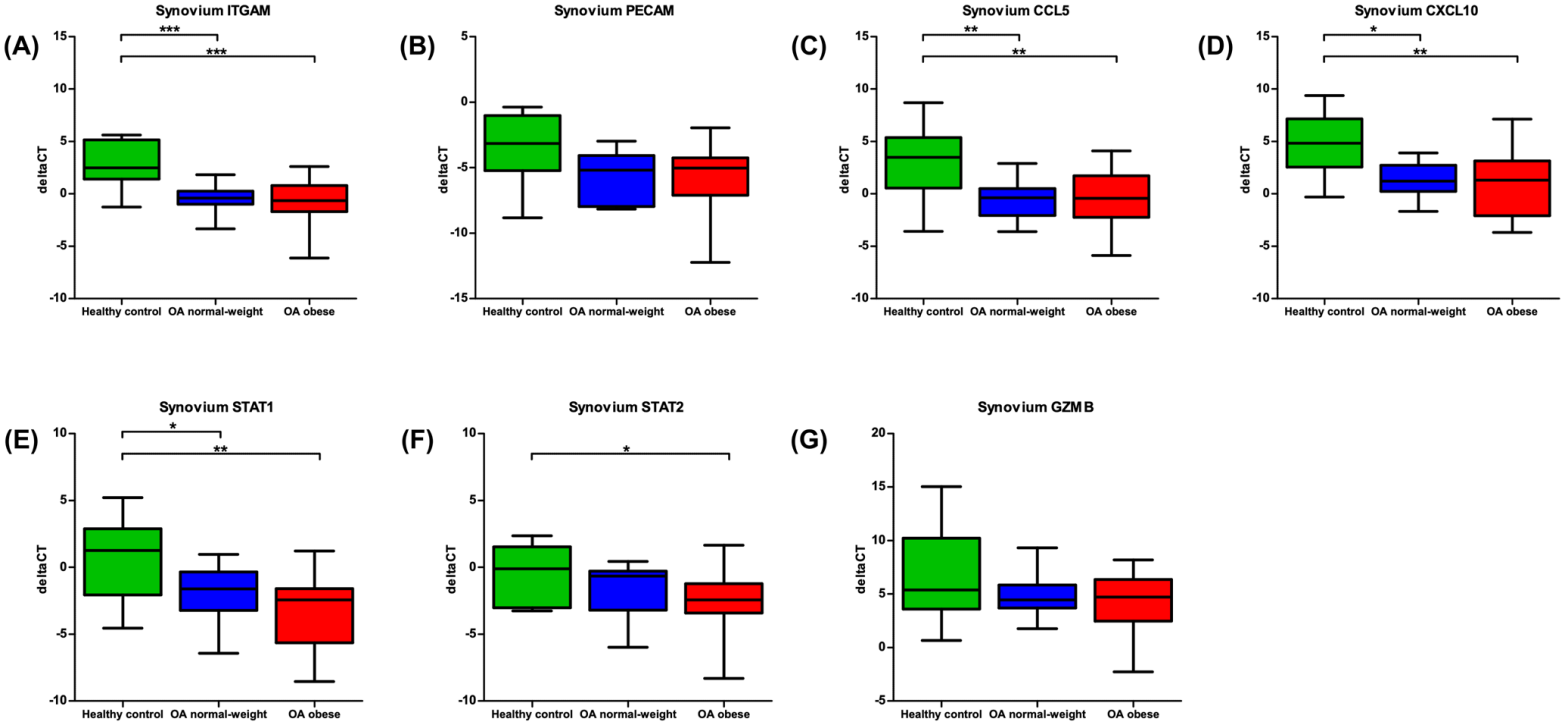
Synovial tissue gene expression profiles in healthy controls and osteoarthritis (OA) patients. Box and whisker plots display ΔCt values (y‐axis; normalised to GAPDH) for (A) ITGAM, (B) PECAM, (C) CCL5, (D) CXCL10, (E) STAT1, (F) STAT2, and (G) GZMB, quantified by qPCR. The x‐axis compares groups: Healthy Controls (synovium from neck‐of‐femur fracture patients), OA Normal‐Weight, and OA Obese. Lower ΔCt reflects higher gene expression. Whiskers span the full data range, boxes represent the IQR (25th–75th percentiles), and the central line marks the mean. Significance thresholds: “*”*p* < 0.05, “**”*p* < 0.01, “***” *p* < 0.001 (One‐way ANOVA with Tukey post hoc test for normally distributed data; Kruskal–Wallis with Dunn's post hoc test for not‐normally distributed data).

Quantitative RT‐qPCR analysis of synovial tissue revealed significant alterations in PDAC‐associated gene expression between healthy controls (neck‐of‐femur fracture patients) and osteoarthritis (OA) patients (Figure 7). ITGAM expression was markedly elevated in both normal‐weight OA patients (mean ΔCt difference = 3.339, *p* < 0.0001, 95% CI: 1.431–5.247) and obese OA patients (3.396, *p* < 0.0001, 1.552–5.239) compared to controls, with no significant difference between BMI groups in OA (0.056, *p* > 0.05). Similar patterns were observed for CCL5 (normal‐weight OA: 3.963, *p* = 0.0015; obese OA: 3.770, p = 0.0015), CXCL10 (normal‐weight OA: 3.408, *p* = 0.0039; obese OA: 3.881, *p* = 0.0039), and STAT1 (normal‐weight OA: 2.586, *p* = 0.0019; obese OA: 3.897, p = 0.0019), with no intergroup differences among OA patients. STAT2 showed significant upregulation only in obese OA patients versus controls (*p* = 0.0346), whereas PECAM and GZMB expression remained unchanged across all groups.

**FIGURE 8:**
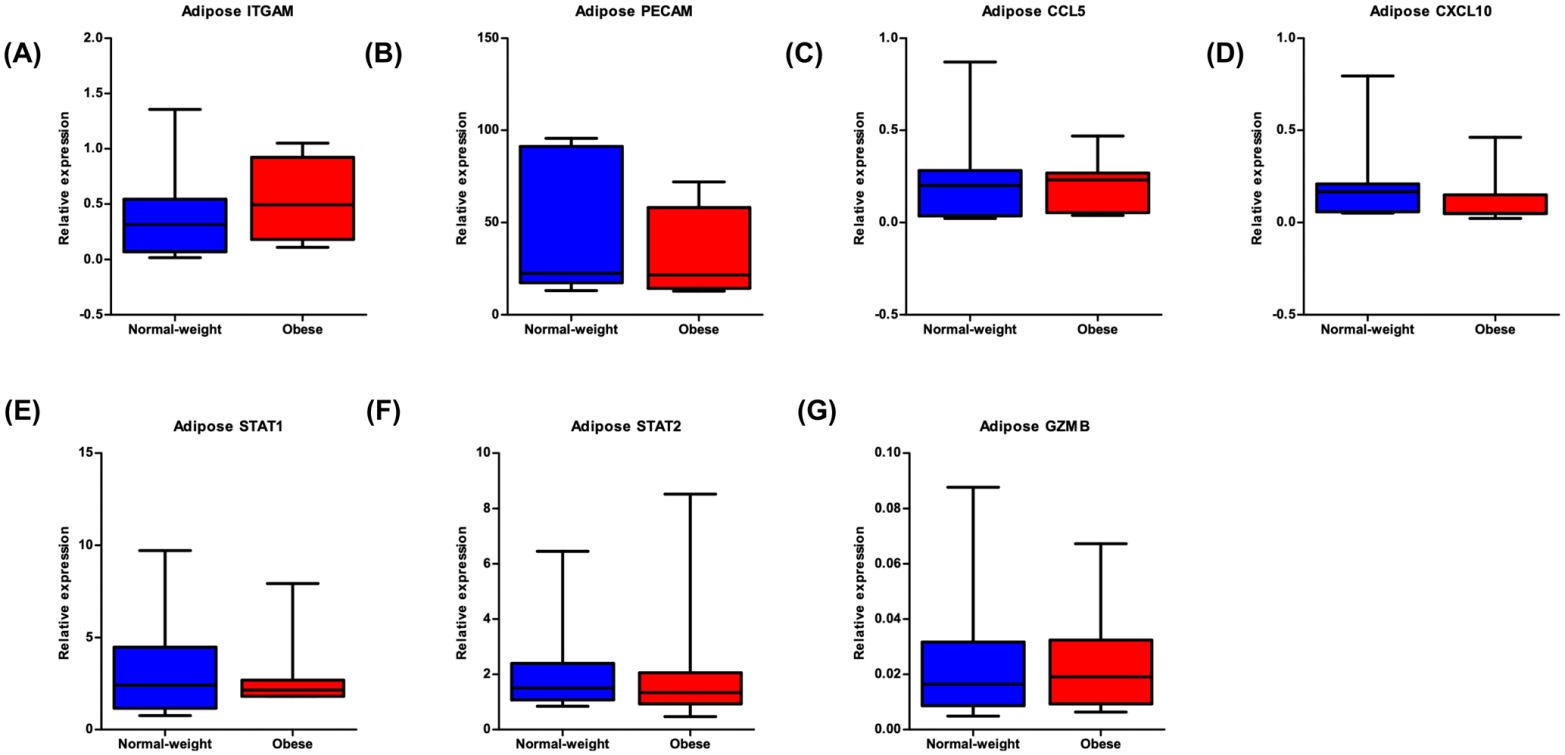
Adipose tissue gene expression profiles in osteoarthritis (OA) patients stratified by BMI. Box‐and‐whisker plots display ΔCt values (y‐axis; normalised to GAPDH) for qPCR‐measured genes: (A) ITGAM, (B) PECAM, (C) CCL5, (D) CXCL10, (E) STAT1, (F) STAT2, and (G) GZMB. The x‐axis compares the OA Normal‐Weight and OA Obese groups. Lower ΔCt values indicate higher gene expression. Whiskers show the full data range, boxes span the IQR (25th–75th percentiles), and the central line denotes the median. Significance thresholds: *p* < 0.05, *p* < 0.01, *p* < 0.001 (*t*‐tests or Mann–Whitney tests if not normally distributed).

In contrast to synovium, subcutaneous adipose tissue exhibited no significant differential expression between normal‐weight and obese OA patients for any tested genes (Figure 8). *ITGAM* showed a non‐significant trend toward higher expression in obese patients (mean ΔCt difference = 0.910, *p* = 0.3417, 95% CI: −1.092 to 2.912). All other genes—*PECAM1* (*p* = 0.7012), *CCL5* (*p* = 0.9520), *CXCL10* (*p* = 0.2622), *STAT1* (Mann–Whitney *p* = 0.9015), *STAT2* (*p* = 0.6758), and *GZMB* (*p* = 0.9167)—demonstrated comparable expression levels between groups. This tissue‐specific pattern suggests synovium may more sensitively reflect inflammatory states relevant to PDAC progression.

## 4 Discussion

**FIGURE 9:**
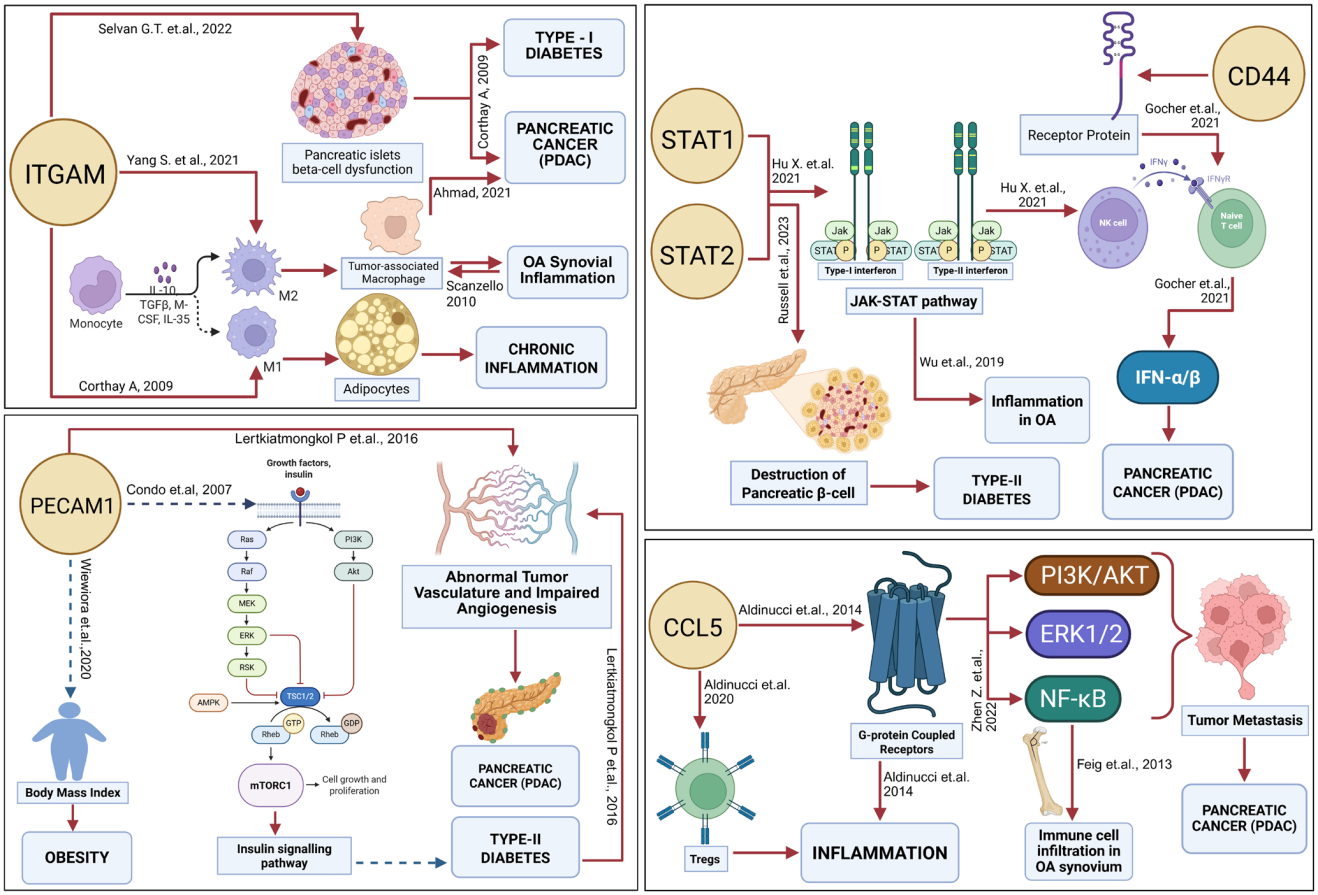
Proteogenomic insights and pathway study of genes of interest showing how each gene contributes to pancreatic cancer and metabolic disorders like Diabetes, Obesity and Inflammation alongside osteoarthritis, by triggering different pathways. Solid red lines signify upregulated expressions and dotted blue lines signify downregulated expressions.

This study was designed to leverage previously validated PDAC recurrence‐associated genes as biological anchors to identify conserved, therapeutically actionable immune‐metabolic signalling pathways that link systemic metabolic dysfunction with pancreatic cancer progression and recurrence, and our cross‐species integrative analyses successfully sketches such pathway‐level associations. The upregulation of key genes like ITGAM, PECAM1, CCL5, STAT1, STAT2, and CD44 in both human and mouse models of obesity highlights their involvement in inflammatory and immune processes that are central to both metabolic dysfunction and cancer progression. These genes, particularly ITGAM and CCL5, are known to modulate immune cell recruitment, inflammation, and lipid metabolism, processes that are upregulated in obese states and contribute to a pro‐tumorigenic environment in PDAC. The single‐cell RNA‐Seq analysis further reveals that clusters within the pancreatic tumour microenvironment, especially Cluster 9, are enriched with immune and inflammatory markers, suggesting a distinct cellular population that may drive tumour recurrence through immune‐mediated mechanisms [39]. The pathway enrichment analysis aligns with these findings, indicating that immune and inflammatory pathways are significantly involved in the tumour microenvironment of PDAC, with pathways like Rheumatoid arthritis, Type I diabetes mellitus, and Inflammatory bowel disease being prominently enriched. These pathways are known to be associated with chronic inflammation and autoimmune responses, which are upregulated in metabolic disorders [42] and could contribute to the immune evasion and recurrence of PDAC post‐resection. The protein–protein interaction network analysis provides further evidence of the critical roles of ITGAM, STAT1, and IL1B in regulating metabolic and inflammatory pathways across both human and mouse models, underscoring the conserved nature of these processes in PDAC recurrence. The cross‐species translational overview suggests that targeting these key metabolic and immune pathways could be a viable strategy to prevent PDAC recurrence and improve outcomes after surgical resection [55, 56]. By modulating the activity of these genes and their associated pathways, it may be possible to reduce the chronic inflammation and immune dysregulation that drive PDAC recurrence, thus minimising fatality in patients post‐surgery. These findings advocate for the integration of metabolic and immunological interventions in the treatment regimen of PDAC, particularly in patients with underlying metabolic disorders, to improve survival rates and reduce the likelihood of cancer recurrence. ITGAM, which plays a crucial role in monocyte differentiation and the polarisation of M1 macrophages [43] is implicated in driving chronic inflammation within the tumour microenvironment. In the context of metabolic disorders like diabetes and obesity, the upregulation of ITGAM enhances this inflammatory response [44], promoting a pro‐tumorigenic environment. This occurs through interactions between tumour‐associated macrophages (TAMs) and adipocytes, which are abundant in obese tissues [44]. The results of this study suggest that ITGAM‐mediated pathways could be pivotal in the development of PDAC, particularly in obese patients, by fostering an environment that supports tumour growth and immune evasion [56]. PECAM1 is another gene of interest, primarily known for its role in maintaining vascular homeostasis and endothelial cell function [45]. Its dysregulation, particularly in the context of obesity and diabetes, is linked to impaired angiogenesis and abnormal tumour vasculature in PDAC [46]. This is further compounded by the dysregulation of the PI3K/AKT/mTOR pathway [46, 47], commonly observed in obesity, which promotes cell proliferation and survival in the tumour (Figure 9). The study indicates that the abnormal signalling through PECAM1 could be a key factor in the vascular abnormalities seen in PDAC, thereby contributing to the aggressive nature of the disease and its recurrence after resection. The study also highlights the significance of STAT1 and STAT2 transcription factors that are central to type I interferon signalling [47]. In the context of diabetes, these factors are often dysregulated, leading to the destruction of pancreatic β‐cells and a compromised immune response [48, 55]. This creates a permissive environment for PDAC development and progression, as the immune system is less capable of mounting an effective response against tumour cells [51]. Additionally, the link between STAT1/STAT2 signalling and inflammation‐driven cancer progression highlights the potential of these pathways as therapeutic targets, particularly in patients with underlying metabolic disorders [47, 55]. CD44, a cell surface receptor involved in cell adhesion and migration, is another critical gene identified in this study. CD44 interacts with immune cells to mediate responses to interferons, which are crucial in the context of both diabetes and PDAC. Its upregulation in PDAC underscores its dual role in promoting inflammation and facilitating tumour metastasis [55]. The study suggests that targeting CD44 could disrupt these processes, potentially reducing the metastatic spread of PDAC in patients with metabolic disorders [55]. CCL5, a chemokine involved in immune cell recruitment and chronic inflammation, also plays a significant role in the inflammatory tumour microenvironment of PDAC. Through its interactions with G‐protein coupled receptors and the downstream activation of the PI3K/AKT and NF‐κB pathways [22, 47] CCL5 drives tumour metastasis. The study highlights the importance of CCL5 in linking chronic inflammatory conditions like IBD with PDAC progression, suggesting that modulating its activity could be a valuable strategy for managing PDAC, particularly in patients with concurrent inflammatory or metabolic disorders [55]. Overall, this study offers a comprehensive understanding of how metabolic disorders contribute to PDAC recurrence by activating pathways related to inflammation, immune dysregulation, and abnormal cellular signalling [57]. The insights gained suggest that targeting these pathways could offer new therapeutic strategies for managing PDAC [52], especially in patients with underlying metabolic conditions. By focusing on the molecular pathways of PDAC in the context of obesity, diabetes, IBD, and inflammation, this study provides a foundation for the development of targeted therapies that could improve patient outcome [56].

## 5 Limitations

A limitation of this study is the use of depot‐specific transcriptional heterogeneity of adipose tissue (e.g., visceral versus subcutaneous), which may influence gene expression effect sizes and partially account for the observed differences between Figures 2 and 8. Nevertheless, these findings should be interpreted as reflective of systemic immune‐metabolic dysregulation, enriched for activated myeloid and immune signalling pathways that are also implicated in obesity and diabetes‐associated inflammatory states. Although OA synovium does not recapitulate the pancreatic tumour microenvironment, the observed concordant upregulation of genes such as ITGAM, CCL5, and STAT1 across OA‐synovium, adipose tissues, and PDAC datasets suggests the presence of shared inflammatory transcriptional programs across chronic inflammatory conditions. Hence, future studies incorporating additional metabolically relevant tissues and longitudinal patient cohorts will be required to further refine these associations.

## 6 Conclusion

The combined analysis of high expression levels of ITGAM, PECAM1, CCL5, STAT1, STAT2, and CD44 in both metabolic disorders and PDAC recurrence, unsupervised learning in single‐cell expression profiles, involvement in key signalling pathways, and complex protein interaction networks collectively highlight these genes as promising therapeutic targets. The convergence of these factors suggests that these genes are not only markers of this disease but also active participants in the pathology of PDAC and its association with metabolic disorders [54]. Moreover, the conservation of these pathways across species reinforces the utility of mouse models in studying the metabolic influences on PDAC and supports the translation of these findings to human disease. Targeting these genes and their associated pathways can hence offer new therapeutic strategies that address both the malignant and metabolic components of the disease. Since PDAC recurrence is highly fatal, targeting these therapeutic markers in patients with metabolic disorders has the potential to increase longevity of survival in patients.

## Author Contributions

D.N.: data curation, conceptualization, formal analysis, investigation, methodology, software, validation, visualization, writing – original draft, writing – review and editing. C.D.: experimental validation, data analysis, writing – review and editing. J.P.: experimental validation, writing – review and editing. S.S.: writing – review and editing. S.W.J.: funding acquisition, validation, writing – review and editing. A.A.: conceptualization, funding acquisition, methodology, administration, supervision, validation, writing – review and editing.

## Funding

The authors declare that financial support was received for the research, authorship, and/or publication of this article. This work was supported by research funding from the Medical Research Council (MR/W026961/1) and Versus Arthritis (21530, 21812). The study was performed at the National Institute for Health and Care Research (NIHR) Birmingham Biomedical Research Centre (BRC).

## Disclosure

Methodology statement: All methods were carried out in accordance with relevant guidelines and regulations.

## Ethics Statement

Ethical approval was granted by the UK National Research Ethics Service (NRES 17/SS/0456, 16/SS/0172, and 14/ES/1044), and human tissues were collected from participants undergoing joint replacement surgery following their written consent at the Royal Orthopaedic Hospital, Birmingham (United Kingdom) and Russell's Hall Hospital, Dudley (United Kingdom). Although mouse data were used in the study, no live animals were involved, as the data were obtained from publicly available datasets.

## Conflicts of Interest

The authors declare no conflicts of interest.

## Supporting information


**Figure S1:** PRISMA Workflow for identifying GEO Datasets.

**Figure S2:** Genes involved in individual human enriched pathways (Enrichr Database).

**Figure S3:** Individual Networks for each Gene of Interest (Human Protein Atlas) (A) ITGAM, (B) PECAM1, (C) CCL5.

**Figure S4:** Individual Networks for each Gene of Interest (Human Protein Atlas) (A) STAT1, (B) STAT2, (C) CD44.

## Data Availability

The datasets analysed during the current study are publicly available in the NCBI Gene Expression Omnibus (GEO) repository. The accession numbers for the datasets used are: GSE246221—Liver adipose tissue (Human). GSE229053—Pancreatic acinar cells (Mouse). GSE263644—Cardiovascular system‐associated adipose tissue (Human). GSE225224—Peripheral tissue (Mouse). GSE212966—Pancreatic cancer single‐cell RNA‐Seq (Human). These datasets can be accessed at https://www.ncbi.nlm.nih.gov/geo/ using the respective accession numbers.
