# Supplementary figures and images for: Linking Targeted Pancreatic Cancer Genes With Metabolic Disorders: A Cross‐Species Translational Pathway
Source: Cancer Med. 2026 Apr 5;15(4):e71775. doi: 10.1002/cam4.71775 (PMC13051988; doi:10.1002/cam4.71775)

## Slide 1
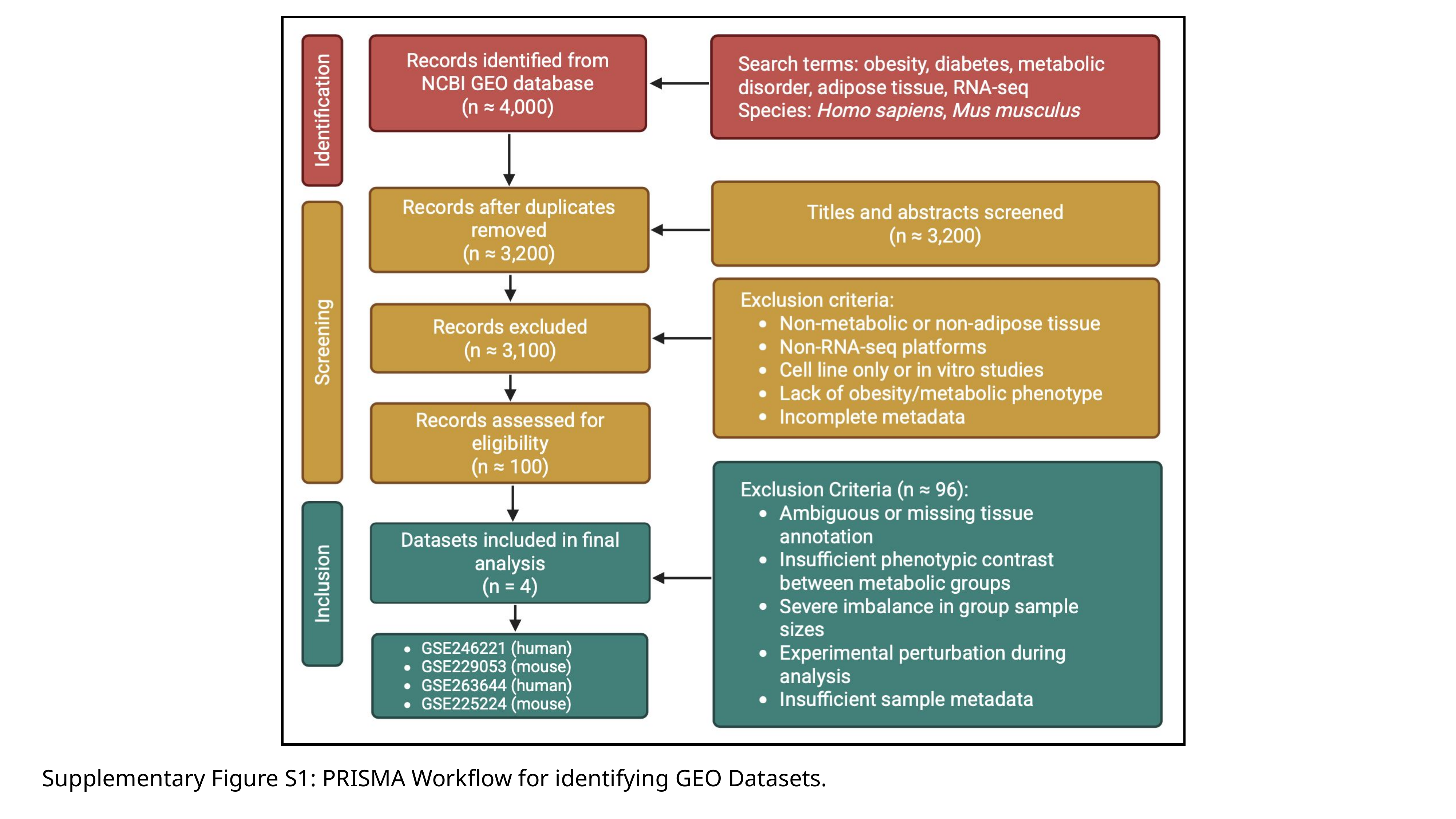

Supplementary Figure S1: PRISMA Workflow for identifying GEO Datasets.

Supplement: Supplementary file 1 — Figure S1: PRISMA Workflow for identifying GEO Datasets. [file CAM4-15-e71775-s002.pptx]
