# Supplementary material for: Linking Targeted Pancreatic Cancer Genes With Metabolic Disorders: A Cross‐Species Translational Pathway
Source: Cancer Med. 2026 Apr 5;15(4):e71775. doi: 10.1002/cam4.71775 (PMC13051988; doi:10.1002/cam4.71775)
Supplement: Supplementary file 2 — Figure S2: Genes involved in individual human enriched pathways (Enrichr Database). [file CAM4-15-e71775-s003.pptx]

## Slide 1
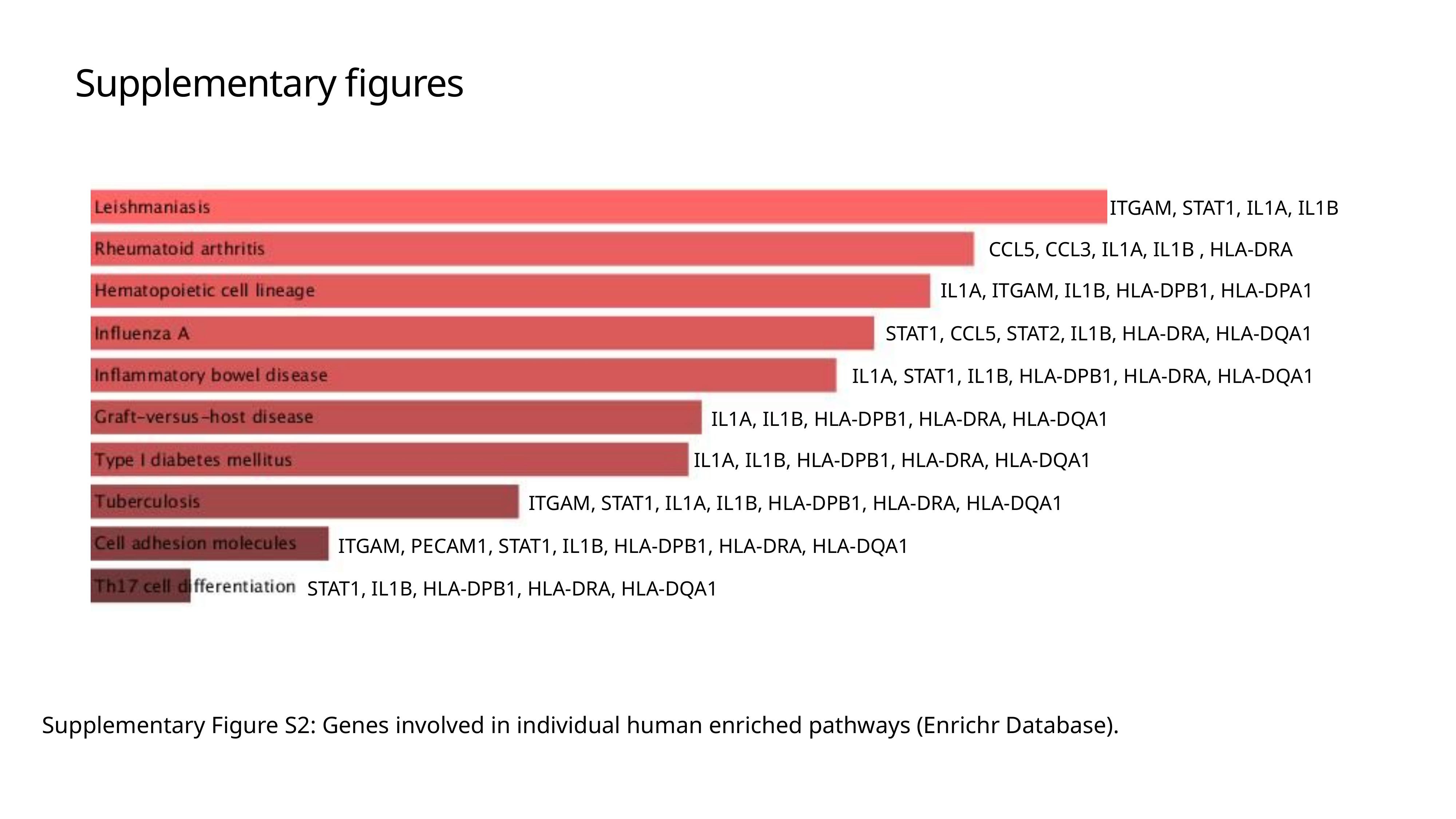

# Supplementary figures
ITGAM, STAT1, IL1A, IL1B
CCL5, CCL3, IL1A, IL1B , HLA-DRA
IL1A, ITGAM, IL1B, HLA-DPB1, HLA-DPA1
STAT1, CCL5, STAT2, IL1B, HLA-DRA, HLA-DQA1
IL1A, STAT1, IL1B, HLA-DPB1, HLA-DRA, HLA-DQA1
IL1A, IL1B, HLA-DPB1, HLA-DRA, HLA-DQA1
IL1A, IL1B, HLA-DPB1, HLA-DRA, HLA-DQA1
ITGAM, STAT1, IL1A, IL1B, HLA-DPB1, HLA-DRA, HLA-DQA1
ITGAM, PECAM1, STAT1, IL1B, HLA-DPB1, HLA-DRA, HLA-DQA1
STAT1, IL1B, HLA-DPB1, HLA-DRA, HLA-DQA1
Supplementary Figure S2: Genes involved in individual human enriched pathways (Enrichr Database).
