# Supplementary material for: Linking Targeted Pancreatic Cancer Genes With Metabolic Disorders: A Cross‐Species Translational Pathway
Source: Cancer Med. 2026 Apr 5;15(4):e71775. doi: 10.1002/cam4.71775 (PMC13051988; doi:10.1002/cam4.71775)
Supplement: Supplementary file 4 — Figure S4: Individual Networks for each Gene of Interest (Human Protein Atlas) (A) STAT1, (B) STAT2, (C) CD44. [file CAM4-15-e71775-s001.pptx]

## Slide 1
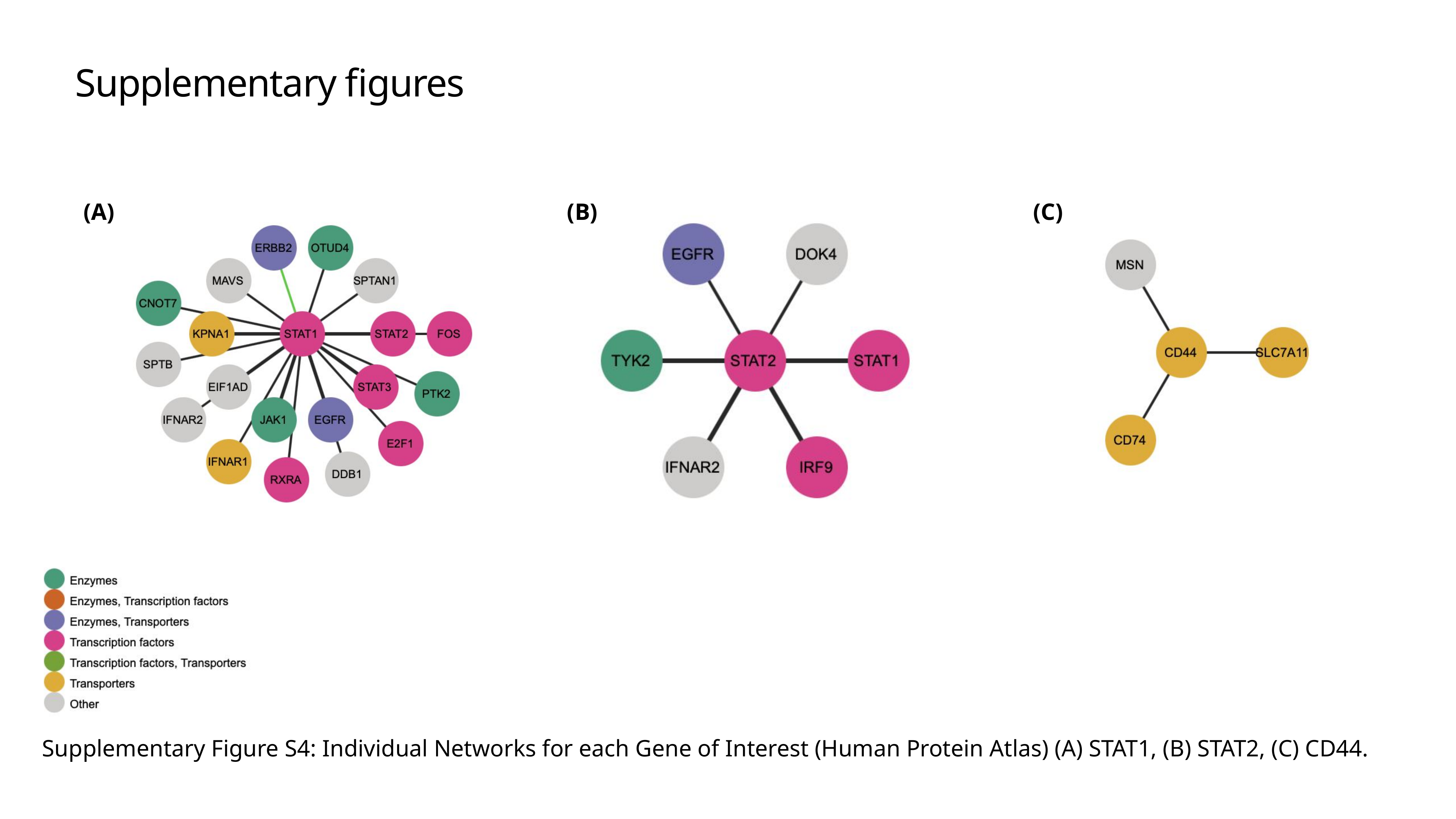

# Supplementary figures
(A)
(B)
(C)
Supplementary Figure S4: Individual Networks for each Gene of Interest (Human Protein Atlas) (A) STAT1, (B) STAT2, (C) CD44.
